# From scarcity to sustainability: Policy pathways for equitable snakebite antivenom access in Africa

**DOI:** 10.1371/journal.pgph.0006490

**Published:** 2026-05-19

**Authors:** Ini Umoh, Ezekiel Boro, Charles McLoughlin, Frank-Leonel Tianyi, George O. Oluoch, Olawale Ajose, Daniel Irowa-Omoregie, Ian Burn, Becky Jones-Phillips, Nicholas R. Casewell

**Affiliations:** 1 Enterprise and Innovation Department, Liverpool School of Tropical Medicine, Liverpool, United Kingdom; 2 Centre for Snakebite Research and Interventions, Liverpool School of Tropical Medicine, Liverpool, United Kingdom; 3 Kenya Snakebite Research and Intervention Centre, Kenya Institute of Primate Research, Ministry of Health, Nairobi, Kenya; 4 Market Access Africa, Geneva, Switzerland; 5 Johns Hopkins Bloomberg School of Public Health, Baltimore, Maryland, United States of America; 6 Faculty of Humanities and Social Sciences, University of Liverpool, Liverpool, United Kingdom; Nasarawa State University, NIGERIA

## Abstract

Snakebite envenoming (SBE) causes substantial mortality and long-term disability in sub-Saharan Africa (SSA), disproportionately affecting rural and economically vulnerable populations. Antivenom is currently the only approved snakebite therapy, yet chronic shortages, fragmented supply chains, high prices, and inconsistent product quality continue to undermine access, exacerbated further by persistent gaps in human resource capacity and health system infrastructure. This article synthesizes existing evidence on antivenom access barriers in SSA and shares feasible policy solutions with a health systems lens grounded in ongoing regional capacity strengthening efforts, World Health Organization (WHO) guidance, and market-shaping experience from other global health initiatives. Although there are multiple bottlenecks along the access continuum, the article focuses on three interlinked challenges: i) underinvested research capacity; ii) fragile and insufficient manufacturing capacity and iii) lack of sustainable financing. Emerging political commitments in countries like Kenya, coupled with expanding research and manufacturing capacity in the region, offer an opportunity to build a resilient regional ecosystem. Learnings from pooled procurement and blended finance in other therapeutic areas indicate potential pathways for stabilizing markets, reducing prices, and incentivizing sustainable production. Strengthening antivenom access in SSA therefore requires a coordinated policy approach anchored in regional research networks, regional manufacturing and blended finance, which collectively offer a viable route to reliable supply, improved affordability, and improved outcomes for tropical snakebite patients.

## Introduction

Snakebite envenoming (SBE) constitutes a devastating yet profoundly neglected public health crisis that has detrimental effects on resource-poor rural communities globally, especially in sub–Saharan Africa (SSA). Annually, an estimated 314,078 snakebites occur in SSA, with some estimates reaching nearly 500,000 cases [1]. This hidden problem claims between 7,331 and 30,000 [2] lives each year in the region, with 97% of these deaths occurring in remote rural areas where prompt access to life-saving treatment remains a challenge. Beyond the immediate fatalities caused by this acute medical emergency, SBE accounts for an estimated 1.03 million Disability Adjusted Life Years (DALYs) annually in SSA, a burden comparable to that of several other neglected tropical diseases (NTDs) [3]. Premature deaths account for 77.2% of this DALY burden [4], with long term disabilities such as amputations and post-traumatic stress disorder negatively impacting survivors and their families [3,5]. The economic toll is equally worrying, disproportionately impacting farmers and herders, who are the backbone of agrarian societies, and are bitten while performing their duties, leading to considerable productivity loss. This incapacitation leads to lost workdays, reduced income, and financial instability, already pushing vulnerable households deeper into debt as treatment costs often necessitate selling valuables, livestock, or land. In seven ASEAN (Association of Southeast Asian Nations) countries, it was estimated that the total costs associated with snakebite amounted to 2.5 billion USD, equivalent to 0.09% of the region’s gross domestic product (GDP) [6], while in SSA, in Rwanda alone, $74 million is said to be lost annually due to treatment costs and unemployment [7].

Antivenoms are currently the only approved therapeutics for the treatment of snakebite envenoming. These region-specific biologic products consist of animal-derived polyclonal antibodies stimulated via the process of venom hyper-immunization. While antivenoms are undoubtedly life-saving therapeutics, these serotherapies have many deficiencies that limit their accessibility in SSA. They: i) are expensive to those in greatest need, ii) are specific to treating bites by only certain snake species resulting in restricted efficacy, iii) consist of animal-derived polyclonal antibodies that frequently cause adverse reactions, iv) have low batch-to-batch consistency and high variability in terms of the concentration of active pharmaceutical components across products, v) have to be delivered in a clinical environment due to intravenous dosing and the need to manage adverse reactions and vi) are reliant on a cold chain. Despite these limitations, antivenoms are recognized by the World Health Organization (WHO) as essential medicines, and while clinical evidence of their efficacy is often lacking due to limited formalized evaluation in robust clinical trials [8,9], antivenoms have been demonstrated to reduce mortality rates to less than 2% [10] and can reduce long-term complications when administered promptly. Despite their relatively high cost (i.e., $48–315 per vial), with several vials often required to effect cure [11], antivenoms have been demonstrated to be a highly cost-effective intervention [12], and in west Africa it costs public payers $83 - $281 to avert 1 DALY [13,14], and one antivenom therapy averts 2.3 DALYs [4]. Given that this cost is well below the threshold of one-time per capita GDP, in most countries in this region, investment in this intervention yields good value for money.

Despite this evidence, millions in SSA unfortunately do not have access to this effective intervention, incurring considerable social, economic, and societal losses. Antivenom is poorly accessible, with availability in some places being as low as 2.5% [15] of the actual need estimated to be met, and often associated with prohibitive costs [4,14], where a single treatment can equate to several months of a subsistence farmer’s income. For a farmer, who earns a typical annual income of $600, and requires multiple vials per treatment plus other hospital costs, this could force them into catastrophic health expenditures or debt [16]. In many cases, affected patients may first seek out traditional healers, a decision often made due to cost and accessibility, and resulting in ineffective outcomes and often severe downstream effects as the result of delaying access to antivenom.

## Systemic barriers to antivenom access

Access to safe, effective, and affordable antivenom is the cornerstone of snakebite treatment in Africa, yet the access pathway from discovery to delivery can be improved at multiple points. The effectiveness, availability, and affordability of safe antivenoms are also deeply intertwined with the capacity of the health systems responsible for delivering them. Weak infrastructure, shortages of trained health workers, long distances to treatment facilities, and inadequate emergency care capabilities all contribute significantly to the preventable deaths and disabilities caused by snakebites [17,18].

While constraints exist across the entire continuum from research to distribution and clinical care, this article focuses on three interlinked bottlenecks in Africa that are critical to improving population-level access and health outcomes: underinvested regional research capacity, fragile and insufficient manufacturing capacity, and lack of sustainable financing.

**Underinvested regional research capacity:** Across Africa, there are only a small number of established research clusters active on regional snakebite. Kenya is a well-known exemplar with operations spanning venom collection, pre-clinical testing of antivenoms and expertise in clinical management [19]. There are also research sites/clusters in Benin, Cameroon, Ghana, Guinea, Namibia, Nigeria and South Africa. These centers have advanced research and innovation, and they continue to contribute expertise and local contextual knowledge. However, there remains an opportunity to increase their impact as centers of excellence in snakebite research and development (R&D). A survey across 15 African countries published in 2023 [20] revealed that all survey stakeholders were broadly interested in establishing research and policy hubs for clinical and public health research to overcome snakebite evidence-based decision-making barriers. Sustainable antivenom access in Africa will require R&D capacity-building efforts beyond clinical and public health research to more comprehensive, end-to-end capabilities, especially in underinvested areas. However, persistent barriers like limited funding, alongside lack of robust data and limited interest from policymakers remain [20].

A stronger research ecosystem is therefore essential to drive knowledge generation critical for: i) identifying snakebite hotspots within countries to ensure optimal use of limited antivenom resources, ii) evaluating the cost of snakebites to local populations and the cost effectiveness of antivenoms to the wider health system to build a stronger investment case, and iii) building internal capabilities required to manufacture, preclinically and clinically evaluate, and achieve regulatory approval of regionally safe and effective local antivenom products.

**Fragile and insufficient manufacturing capacity**: Antivenom supply in SSA is precariously dependent on a few international manufacturers and a single regional producer, South African Vaccine Producers (SAVP) [7]. SAVP’s polyvalent antivenom is widely used across southern Africa but experiences frequent disruptions in production. In recent years, shortages of equine plasma [21] coupled with frequent power challenges [22], have led to limited production, which in turn has triggered acute supply crises [23]. The consequences of withdrawal or temporary unavailability of effective products that are solely relied upon can be disastrous, with several well documented examples describing dramatic increases in case fatality rates when geographically inappropriate products are procured in response to standard of care antivenom unavailability [24,25]. The withdrawal of several major manufacturers from African markets over the past two decades [26,27] driven by low profitability, unpredictable demand, and high production costs has further destabilized supply. This is exacerbated by the structural dependence on imports [28], with almost all of Africa’s antivenom now manufactured outside of the continent, while Africa produces less than 30% of its essential medicines [29], further highlighting the wider reliance on imported medicines. The COVID-19 (coronavirus disease 2019) pandemic demonstrated the fragility of this dependence: border closures and supply chain disruptions caused shortages across multiple essential commodities, including antivenoms [28,29].

Even when production capacity and supply exist, quality deficiencies undermine therapeutic effectiveness and patient safety across the region. Some antivenoms marketed in SSA fail to adequately neutralize venoms from local snake species, rendering them ineffective [8]. Also, SAVP’s products have not yet been approved by WHO’s formal risk–benefit evaluation of antivenoms, which limits their regulatory recommendation for wider regional procurement [30,31]. The lack of regional quality control laboratories capable of conducting potency testing, coupled with inadequate pharmacovigilance systems reinforces a market with products that are not best suited to the local problem.

These supply and quality issues are linked: quality failures nullify supply gains, as products not effective for regional snakes contribute little to no therapeutic access improvements regardless of volume produced. This further worsens an inequitable antivenom access situation, where production decisions are shaped by unpredictable demand, intermittent procurement, and limited regulatory oversight, conditions that deter long-term investment in quality improvements or scale.

**Lack of sustainable financing**: Funding constraints augment existing supply-side challenges. Between 2007 and 2024, only $156 million USD was invested in R&D for snakebite envenoming compared with $13 billion for malaria and $28 billion for HIV/AIDS (Human Immunodeficiency Virus/Acquired Immune Deficiency Syndrome) over the same period [32]. Also, this neglected disease remains heavily donor-dependent, with just two funders accounting for an estimated 65% of global funding in 2024 [18] and available funding is often concentrated on research rather than implementation, limiting the resources needed to strengthen manufacturing, delivery systems and scale proven interventions.

Recent global cuts in development assistance [33] threaten to further divert attention and resources towards higher-profile transmissible diseases that align more closely with current donor priorities, leaving snakebite, typically an accidental and less visible condition, more marginalized. This is further compounded by inadequate government investments. Although the 2018 World Health Assembly resolution on snakebite urged member states to improve access to treatment by mobilizing national resources [34], a 2025 review of NTD masterplans from 15 African countries suggests there is limited practical implementation of integrated SBE activities in national NTD programmes [35].

Few countries in SSA procure antivenoms for their populations, with much of the supply burden falling on the private sector and patients who bear the healthcare costs through out-of-pocket payments. This leads to unfavourable incentives embedded within health system procurement and practice across the region. Procurement processes have defaulted to price-based tendering that prioritizes the least expensive available products irrespective of clinical effectiveness or safety profiles. This race to the bottom procurement strategy creates a market environment where manufacturers are not incentivized to invest in product improvement, such as improved potency against regional snake species, increased antibody content or enhanced safety standards, as these quality improvements command no price premium and may actually disadvantage suppliers in lowest price tenders. The predictable consequence is a vicious cycle: ineffective antivenoms that reduce clinical confidence, prompting physicians to administer multiple vials empirically to achieve therapeutic effect, thereby wasting scarce resources. If the current trajectory continues, access to antivenoms in SSA will remain inequitable and prohibitively expensive for many communities. Failure to address these bottlenecks will mean that preventable deaths and disabilities from snakebite will not be averted, health systems will face repeated crises of stockouts, and economic losses will continue. Projections suggesting that parts of the continent may see an increase in venomous snake species richness and snakebite in the coming years because of climate change [36] may further exacerbate this situation. Ultimately, in the absence of sustainable financing models, the current trajectory of investments is unlikely to meet the real scale of need or enable the WHO to meet their targets to halve snakebite deaths and disabilities by 2030 [37].

## Policy pathways to a sustainable antivenom ecosystem in Africa

Addressing and overcoming these barriers requires coordinated, systemic action across R&D capacity strengthening, manufacturing, and financing. We present a three-pronged strategy grounded in the realities of African health systems and supported by existing evidence, policy recommendations, and shared learnings from the global experience of tackling other diseases like COVID-19, HIV, malaria, and tuberculosis.

### 1. Build and network regional research hubs

A sustainable antivenom ecosystem relies on strong R&D networks that integrate basic, pre-clinical, clinical and public health scientific expertise. A hub and spoke research network of regional centers of excellence across Africa, supporting activities such as i) venom collection and characterization including the provision of regional venom standards, comprehensive venom profiling, and species distribution mapping ii) toxinological and immunological research to devise optimal immunogens required for regional antivenom production, iii) robust pre-clinical and clinical evaluation of antivenoms to support regulatory decision making, and iv) public health (epidemiology, health economics, operational, policy, etc.) research is key to ensure a steady pipeline of antivenom interventions to address the inequitable access issues in Africa.

The network could operationally thrive by concentrating specialized expertise and infrastructure within a set of regional hubs, linking these hubs across countries, and connecting them to industry partners and funders. Dedicated programs for training, capacity strengthening, and mentorship will be key to developing a continuous pipeline of skilled researchers capable of maintaining and advancing the network over time.

A similar regional approach was previously employed by the EchiTAb Study Group, which resulted in the provision of two new antivenoms to west Africa, supported by the Nigerian Federal Ministry of Health and enabled by north-south and south-south academic-commercial collaborations [38,39], while the UK National Institute for Health and Care Research (NIHR)-funded African Snakebite Research Group project sought to build capacity for snakebite research in several African countries, leading to the formation of regional snakebite research and intervention centers in Nigeria, Kenya and eSwatini [40].

Sustainable investments from both African governments and donors in R&D, manufacturing and delivery capacity and infrastructure will be required to maintain the capabilities and scale of these research hubs and networks across the continent [41]. Earlier similar SBE network initiatives, while commendable, ultimately fell short of addressing systemic access challenges because funding was reduced or withdrawn.

### 2. Strengthen regional manufacturing capacity

Regional manufacturers like SAVP were historically critical for Southern Africa’s antivenom supply but now require targeted investments and support to modernize their manufacturing facilities to meet WHO’s quality benchmarks and stabilize supply of equine plasma and other critical manufacturing infrastructure, including energy security. These enhanced manufacturing processes, priorities and targets must be underpinned by WHO’s antivenom target product profiles and antivenom risk-benefit assessment procedures [10,30] which collectively provide clear guidance and regulatory assurance support for manufacturers. Manufacturing capacity strengthening support for other potential local manufacturers must happen concurrently to avoid the risk of sole dependence on one SSA regional manufacturer, particularly given past challenges as the result of vulnerabilities associated with single points of failure [42]. This could be facilitated through: i) technology transfer partnerships with established global manufacturers, e.g., focused on facility establishment, process optimization and validation appropriate for local scale, ii) building public-private and/or public/government-led manufacturing facilities, such as those well-established in parts of Latin America [43], or iii) scaling existing snakebite research and intervention centers in countries like Kenya and Nigeria into venom standard, fill-finish and preclinical testing nodes as part of global manufacturing networks. Encouragingly, a few countries, including Kenya have signaled political willingness to develop and manufacture antivenoms in country [44]. Under the Kenyan Ministry of Health, the Kenya Institute of Primate Research (KIPRE) has recently, i) established the Kenya National Antivenom Quality Control Laboratory to provide independent preclinical testing to inform in country regulatory approval and procurement decision making [45], and ii) has plans to launch a locally made antivenom for the region [46]; this initiative could further strengthen and catalyze the wider sub-regional and regional SBE R&D capacity if appropriately supported. The WHO mRNA (messenger ribonucleic acid) technology transfer program [47] established by WHO, Medicines Patent Pool and COVID-19 Vaccines Global Access (COVAX) for COVID-19 vaccine manufacturing, demonstrates the feasibility and value of establishing and implementing coordinated north-south and/or south-south technology transfer models, which could be of great value for snakebite and wider antivenom production.

### 3. Create sustainable and innovative financing mechanisms

Sustained investment in technology transfer, regulatory systems strengthening, and locally led governance and financing will be essential to ensure that both R&D and manufacturing capacity remain viable. Investments to strengthen R&D and manufacturing capacity should go in tandem with investments to address existing health system infrastructural and capacity deficits in SBE (including first aid and anaphylaxis) clinical management, human resourcing, training curricula, treatment protocols, Instructions for Use (IFU) materials, etc.

Innovative financing should be considered as a major enabler, as the uptick in global aid retrenchment makes traditional grant funding an unsustainable solution for the arduous task of scaling antivenom production and access. To secure short term supply and better prices, while the region builds production capacity, demand aggregation in the form of pooled procurement layered with advanced market commitment (AMCs) can improve supply and reduce prices by giving manufacturers credible forward revenue streams [48–50]. Data analyses from several low and middle-income countries—including Senegal, South Africa, Tunisia and Zambia—show that pooled procurement can reduce medicine prices by an average of 15% across product groups such as HIV antiretrovirals and tuberculosis medicines [49,51]. In 2025, the Africa CDC (Centres for Disease Control and Prevention) launched the African Pooled Procurement Mechanism in ten pilot countries, with the goal of achieving 30–40% price reduction and 90% availability for ten priority health products [52–54]. Extending this mechanism to include product classes for neglected diseases such as SBE could be highly impactful. By consolidating demand across countries into a unified market, the mechanism helps overcome common barriers such as limited technical and financial capacity among individual buyers and ensures greater homogeneity in purchasing requirements—factors that significantly enhance the likelihood of procurement success [55]. In turn, this mechanism could also foster the formation of a strategic rotating stockpile—funded nationally, sub-regionally or regionally (for instance, by the Africa CDC) through a snakebite stockpile fund—which aligns with WHO’s strategy to stockpile WHO-recommended antivenoms [56]. This strategic stockpile could also stabilize the market through risk-sharing and increase in trust of demand amongst high-quality suppliers. Concurrent with a pooled procurement instrument, price setting for AMCs should consider the nuances of this disease problem (i.e., whether there is shared snake species distribution and cross-snake species antivenom efficacy) in addition to the countries’ willingness to pay, and to reduce the reliance on a single producer, AMCs should be obtained from multiple producers.

Pooling resources with nearby regions is more efficient, but it may not provide a durable long-term financing solution. To mitigate this, a dedicated regional vehicle mixing donor grants for CAPEX (capital expenditure), concessional loans from development finance institutions, and subordinated private capital is needed to finance venom banks, preclinical efficacy testing facilities, fill-finish sites and Good Manufacturing Practice (GMP) manufacturing hubs [4,57,58]. Though blended finance favors larger, more creditworthy projects [59–61], and antivenom production may not attract substantial capital, this mechanism is useful where concessional grants absorb initial risk and make projects bankable; and public governance can aggregate projects to increase scale and risk diversification [58–61]. The requirement of strong public governance and financial management makes this type of investment a worthwhile option for governments seeking high-impact industrial policy outcomes, and the distribution of obligations among states could potentially benefit low-capacity countries. The potential and scale of economic and societal benefits through job creation and spillovers into other sectors should be weighed when considering the value of this intervention.

## Conclusion

SBE remains a preventable crisis with a cost-effective solution in the form of antivenom, however access to this essential medicine is limited by challenges that span across underinvestment in regional research capacity, limited antivenom production, and poor funding. In the SSA regional context, historical over-reliance on a single in-continent antivenom producer, vulnerability to global shocks such as COVID-19, and donor dependent financing are some of the fragilities of current systems. To secure sustainable access, this paper advocates for three reinforcing strategies: strengthening local research capacity, improving regional manufacturing, and mobilizing a blend of donor, regional, national and innovative financing mechanisms. Together these approaches can transform siloed efforts into a resilient, equitable and sustainable antivenom ecosystem in Africa, to reduce the burden of snakebite across the continent.
